# Preliminary analysis of the role of small hepatitis B surface proteins mutations in the pathogenesis of occult hepatitis B infection via the endoplasmic reticulum stress-induced UPR-ERAD pathway

**DOI:** 10.1515/biol-2022-0951

**Published:** 2025-02-04

**Authors:** Chengrong Huang, Hao Zhang, Jing Wang, Jianfei Li, Qian Liu, Qiyin Zong, Yunyun Zhang, Qin Wang, Qiang Zhou

**Affiliations:** Department of Clinical Laboratory, The Second Affiliated Hospital of Anhui Medical University, Hefei, 230601, China; Department of Clinical Laboratory, Anqing Municipal Hospital, Anqing, 246003, China; Department of Clinical Laboratory, Nanjing Jiangning Hospital, Nanjing, 211100, China; Department of Clinical Laboratory, The Second Affiliated Hospital of Anhui Medical University, 678 Furong Road, Hefei, 230601, China

**Keywords:** occult hepatitis B virus infection, hepatitis B surface antigen, mutation, endoplasmic reticulum stress, ER-associated protein degradation

## Abstract

A growing body of evidence has shown that hepatitis B surface antigen (HBsAg) mutations can influence the occurrence of occult hepatitis B infection (OBI), particularly amino acid substitutions in small hepatitis B surface proteins (SHBs). The mechanistic basis for these results, however, remains unclear. This study was designed to explore the potential impact and mechanisms of OBI-related SHBs mutations on serum HBsAg. Huh7 and HepG2 cells were transfected with plasmids encoding wild-type (WT) or OBI-related SHB mutation-containing sequences, after which a chemiluminescence approach was used to detect HBsAg levels in cell culture supernatants. Western blotting was further used to assess HBsAg and endoplasmic reticulum stress (ERS)-related protein levels in lysates prepared from these cells, while the localization of HBsAg within cells was assessed via immunofluorescent staining. Cells transfected with OBI-related SHB mutation-encoding plasmids exhibited lower supernatant HBsAg levels than cells transfected with WT plasmids. Intracellular and extracellular HBsAg levels in these mutant plasmid-transfected cells were lower relative to those for WT plasmid-transfected cells, and HBsAg accumulation within the ER was detected via immunofluorescent staining in cells transfected with OBI-related SHB mutation-encoding plasmids, ERS-related protein content was also significantly increased in mutant plasmid-transfected cells as compared to those in the WT group. These results suggest that proteins harboring OBI-related mutations may tend to accumulate in the ER, thereby triggering an ERS response and impairing the transcription and translation of HBsAg via the activation of the unfolded protein response and ER-associated protein degradation pathway. These effects ultimately reduce the overall assembly of HBV virions in the ER and their associated secretion.

## 1 Introduction

Occult hepatitis B virus (HBV) infection (OBI) is defined as the persistence of replication-competent HBV DNA in the liver and/or HBV DNA in the blood; meanwhile, the HBV surface antigen (HBsAg) detection was negative by currently available assays. Patients with OBI may exhibit positive anti-HBs, anti-HBe, and/or anti-HBc [1]. HBV in these patients still can be transmitted to other patients through blood transfusions, delivery, hemodialysis, and liver transplantation [2–4]. These patients may also experience viral reactivation in the context of weakened immune system functionality, potentially causing severe symptoms [5,6]. Persistent OBI can also increase the risk of liver cirrhosis and associated cancer [2,7,8]. To date, the exact mechanism of OBI has not been well clarified yet. Emerging studies have shown that mutations in the HBsAg of *HBV* gene, especially the major hydrophilic region (MHR) mutation, can change the antigenicity and immunogenicity of HBsAg, which, in turn, affects the recognition of the virus by immune system. These *HBV* gene mutations can also interfere with the normal release of HBsAg from the endoplasmic reticulum (ER) such that it can accumulate within cells, ultimately contributing to OBI incidence [9–11]. The best-characterized OBI mutations to date include the G145R, G119R, T126A, Q129R, and D144A mutations [10–12].

The ER is the primary organelle in which proteins are synthesized, subjected to post-translational modification, and folded through a maturation process. The majority of transmembrane and secreted proteins are produced by eukaryotic cells that fold and mature within the lumen of the ER. Immature proteins enter the ER in the form of unfolded polypeptide chains [13,14]. The ER stress (ERS) response can be triggered by the accumulation of high concentrations of misfolded or unfolded proteins within the ER [15], engaging a series of regulatory mechanisms that are intended to restore normal ER homeostatic balance. These include the recognition of unfolded or misfolded proteins in the ER and their return to the cytosol wherein they undergo degradation through ubiquitin-proteasome, namely, ER-associated protein degradation (ERAD) [16]. To further aid in the processing of unfolded proteins, cells engage an activating transcription of target genes in response to unfolded proteins, which are called unfolded protein response (UPR). If these mechanisms are insufficient to restore appropriate ER homeostasis, then cells ultimately engage death pathways [17]. The GRP78 protein upregulated by HBV has been shown to play an important role in supporting the survival of infected hepatocytes through the induction of an intermediate level of ERS. By suppressing the replication of HBV, GRP78 maintains the viral load at a relatively low level while establishing a beneficial degree of stress within the cellular microenvironment that is ultimately conducive to viral persistence [18]. The specific role that ERS plays in OBI development, however, has yet to be fully clarified.

The present analyses revealed that HBsAg protein levels were significantly reduced in culture supernatants prepared from cells that had been transfected with plasmids encoding OBI-associated SHB mutations as compared to those from cells transfected with wild-type (WT) SHB plasmids. Immunofluorescent staining further demonstrated that this mutated isoform of HBsAg primarily localized to and accumulated within the ER. SHB mutations were also associated with higher levels of ERS-associated protein expression within transfected cells relative to cells transfected with WT SHBs plasmids. These data indicate that ERS likely plays a major role in the pathogenesis of OBI.

## 2 Materials and methods

### 2.1 Plasmid construction

Plasmids used for this study were constructed by Wanlei Life Sciences (Shenyang) Co., Ltd. WT and mutant SHB plasmids were cloned into the eukaryotic pcDNA3.1(+) vector to generate WT and mutant pcDNA3.1-SHB plasmid constructs. The WT SHB sequence used for this study was the U95551.1 sequence with a 681 bp CDS, and a site-specific mutation kit was used to introduce OBI-associated SHB mutations.


**Ethical approval:** The research related to human use has been complied with all the relevant national regulations, institutional policies and in accordance with the tenets of the Helsinki Declaration, and has been approved by the Ethics Committee of the Second Affiliated Hospital of Anhui Medical University.

### 2.2 Cell culture and transfection

Huh7 and HepG2 cells were provided by the Shanghai FuHeng Cell Bank and cultured in dulbecco’s modified eagle medium (DMEM) containing 10% fetal calf serum and penicillin/streptomycin in a humidified 5% CO_2_ incubator at 37℃. Cells were transfected with the seven constructed plasmids (including empty vector, WT, and OBI-associated SHB mutation-containing plasmids [G145R, T126A, Q129R, G119R, and D144A]) using Lipofectamine 3000 (Invitrogen, CA, USA) and Opti-MEM (Gibco BRL).

### 2.3 HBsAg detection

Extracellular (EC) HBsAg levels were detected in supernatants from transfected cells at 72 h post-transfection with an ARCHITECT i2000SR chemiluminescent immunoassay analyzer. To evaluate intracellular (IC) HBsAg production, supernatant-free cell monolayers were washed three times with phosphate-buffered saline and lysed with radioimmunoprecipitation assay (RIPA) buffer containing protease and phosphatase inhibitors (Beyotime, Shanghai, China). These lysates were then centrifuged for 30 min at 13,000 rpm, and supernatants were isolated as cytosolic extracts for further analysis. HBsAg levels in these extracts were detected with a chemiluminescent immunoassay analyzer.

### 2.4 Western blotting

Proteins were isolated from cells using RIPA buffer supplemented with protease and phosphatase inhibitors, as above, after which these extracts (20 µg/sample) were separated via 10% SDS-PAGE and transferred to polyvinylidene difluoride (PVDF) membranes. These membranes were incubated with 5% nonfat milk and then incubated overnight at 4°C with antibodies specific for the following: HBsAg (1:1,000, bs-1557G; Bioss), GRP78 BiP (1:5,000, ab108615; Abcam), ATF6 (1:1,000, ab227830; Abcam), IRE1 (1:1,000, ab243665; Abcam), XBP1 (1:1,000, ab220783; Abcam), pPERK (1:1,000, DF7576; Affinity), peIF2α (1:1,000, AF3087, Affinity), EDEM1 (1:2,000, DF9502; Affinity), EDEM2 (1:1,000, A17181; ABclonal), and EDEM3 (1:2,000, DF9504; Affinity). Following incubation for 1 h with secondary HRP-conjugated rabbit anti-goat IgG (1:20,000; Zhongshan, Beijing) at room temperature, ECL working solution was used to cover PVDF membrane, X-ray film was used to press PVDF film, develop, fix, and form protein bands, in the dark room.

### 2.5 Immunofluorescent staining

At 72 h post-transfection, cells were fixed for 30 min using 4% paraformaldehyde at room temperature prior to permeabilization for 30 min with 0.1% Triton X-100 and 10% FBS at room temperature. These cells were then probed with polyclonal anti-HBsAg (Bioss, Beijing) and monoclonal anti-KDEL (ER-marker) (Affinity, USA) to evaluate HBsAg and ER co-localization. Secondary donkey anti-goat-Cy3 (Beyotime, Shanghai) and goat anti-rabbit-KDEL (Beyotime, Shanghai) were then used to detect proteins, followed by nuclear counterstaining with 4′,6-diamidino-2-phenylindole (DAPI) (10 μg/mL; Aladdin, Shanghai). Cells were then visualized via confocal microscopy, with HBsAg, the ER, and nuclei, respectively, staining red, green, and blue.

### 2.6 Statistical analyses

SPSS 20.0 (SPSS Inc., IL, USA) was used to analyze all data. Results were generated from a minimum of three independent experiments and are reported as mean ± standard deviation. One-way ANOVAs were used to compare results among groups, and a two-sided *P* < 0.05 was the threshold of significance for this study.

## 3 Results

### 3.1 OBI-related SHB mutations alter IC and EC HBsAg distributions

**Figure 1:**
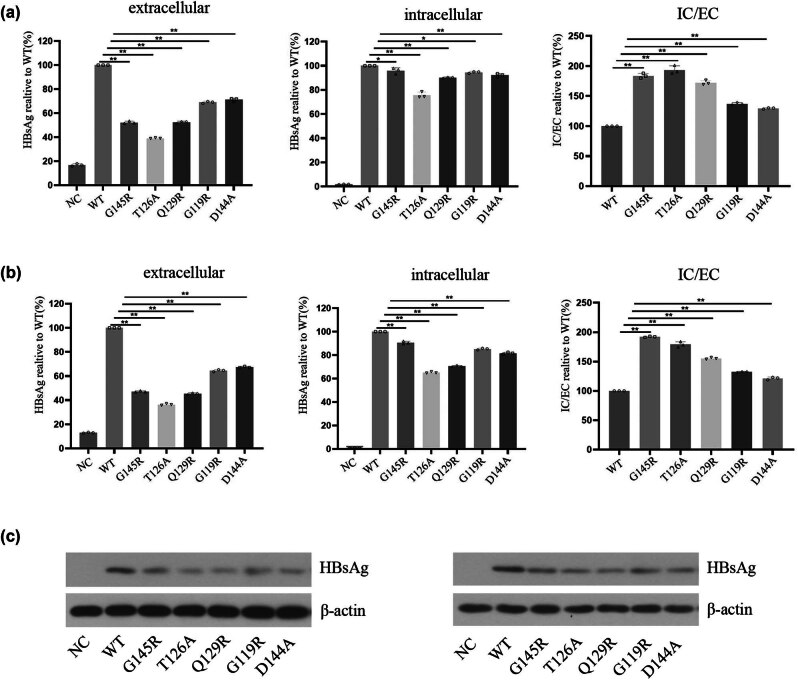
Analyses of IC and EC HBsAg levels in cells transfected with a range of plasmid types. Following pcDNA3.1-SHBs plasmid transfection as indicated, (a) and (b) EC (left) and IC (middle) HBsAg levels were measured by chemiluminescence, and the ratio of IC/EC (right) for HBsAg levels was analyzed in HepG2 cells (a) and Huh7 cells (b). (c) IC HBsAg levels in HepG2 (left) and Huh7 (right) cells were also determined by western blotting. *P < 0.05, **P < 0.001.

For this study, a series of S protein plasmids were constructed including an empty control vector, a plasmid encoding WT HBV-S protein, and a series of HBV-S protein-coding plasmids harboring different OBI-associated mutations (G145R, T126A, Q129R, G119R, and D144A). Huh7 and HepG2 cells were then transfected with these plasmids, and at 72 h post-transfection, the EC and IC HBsAg levels were detected by chemiluminescent assays and western immunoblotting. Relative to those cells transfected with the HBV-S-WT plasmid, those cells that had been transfected with plasmids encoding OBI-associated SHB mutations exhibited significantly lower EC concentrations of HBsAg (Figure 1a and b). IC HBsAg levels were also lower in these mutant plasmid-transfected cells as compared to cells transfected with the HBV-S-WT construct (Figure 1a–c). Then the ratio of IC to EC HBsAg was evaluated and compared. It revealed that the IC/EC ratio for HBsAg of cells that transfected with plasmids encoding OBI-associated SHB mutations exhibited significantly higher than that of cells that transfected with the HBV-S-WT plasmids (Figure 1a and b). This suggests that HBsAg expression and secretion are significantly reduced for cells expressing S protein isoforms harboring these OBI-associated SHB mutations, indicating that these mutations contribute to IC HBsAg accumulation.

### 3.2 Analyses of the subcellular localization of S proteins within cells transfected with plasmids encoding OBI-associated mutations

**Figure 2:**
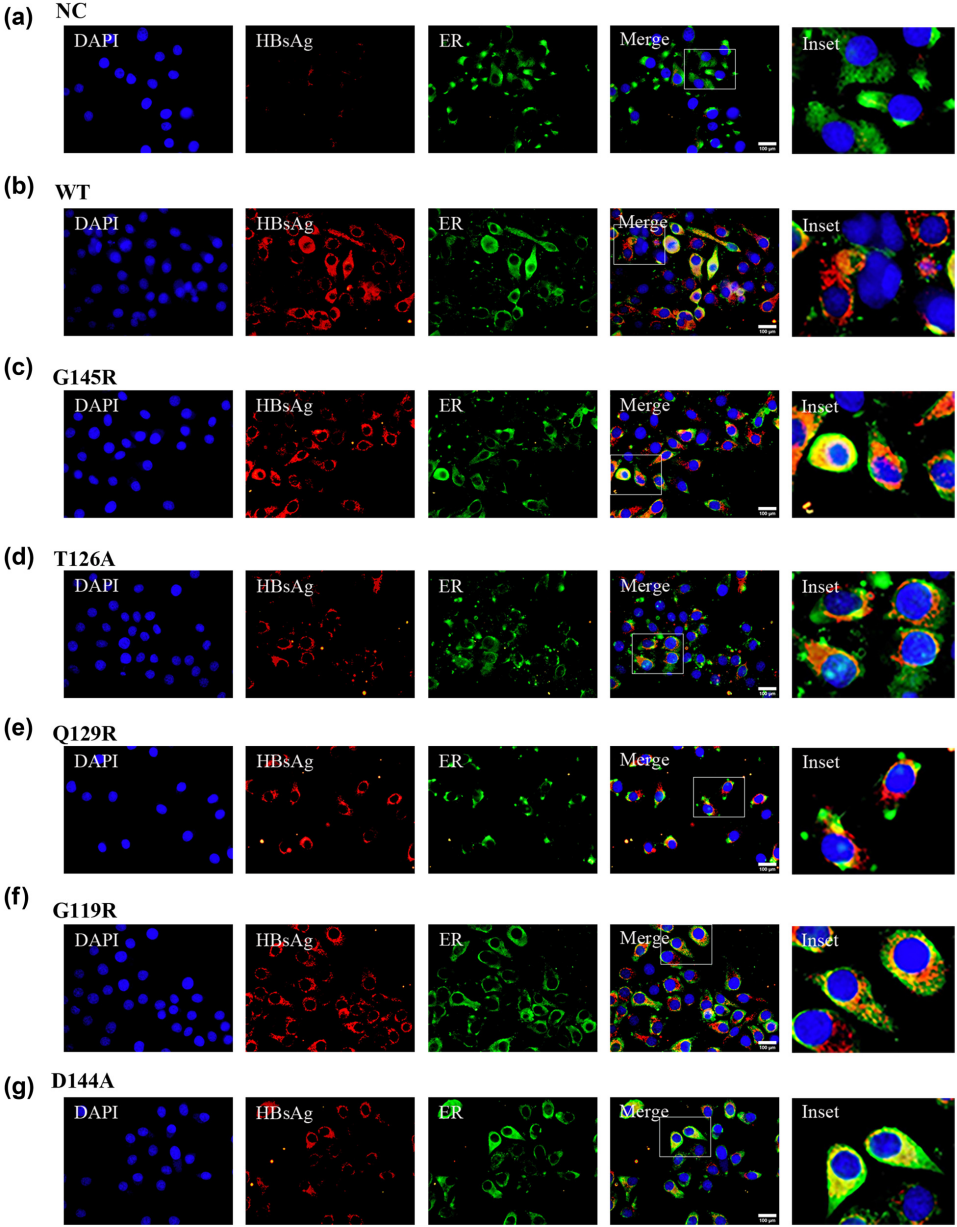
HBsAg localization within HepG2 cells following different plasmid transfection. HBsAg localization was assessed within HepG2 cells following empty vector (a), HBV-S-WT (b), HBV-S-G145R (c), HBV-S-T126A (d), HBV-S-Q129R (e), HBV-S-G119R (f), and HBV-S-D144A (g), plasmid transfection (f). IC HBsAg was stained by Cy3 (green), and ER marker KDEL was stained by FITC (red). The cell nuclei were stained with DAPI (blue). Magnification, ×600. Scale bar 100 µm.

**Figure 3:**
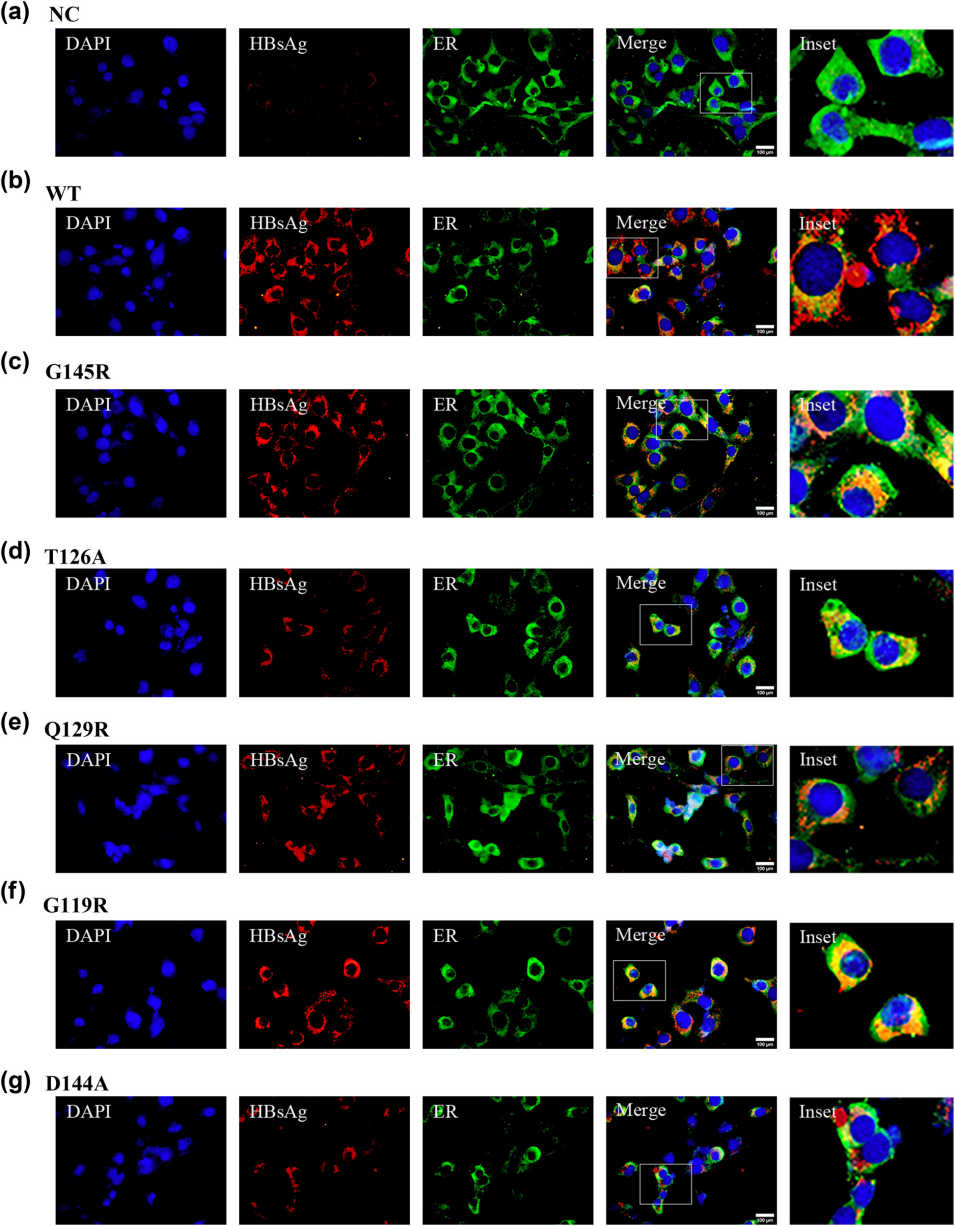
HBsAg localization within Huh7 cells following different plasmid transfection. HBsAg localization was assessed within Huh7 cells following empty vector (a), HBV-S-WT (b), HBV-S-G145R (c), HBV-S-T126A (d), HBV-S-Q129R (e), HBV-S-G119R (f), and HBV-S-D144A (g), plasmid transfection (f). IC HBsAg was stained by Cy3 (green), and ER marker KDEL was stained by FITC (red). The cell nuclei were stained with DAPI (blue). Magnification, ×600. Scale bar 100 µm.

Several preS/S gene mutations have been linked to the abnormal synthesis of this cell surface protein in hepatocytes such that it is retained within the ER [19]. Immunofluorescent staining was conducted to evaluate the S protein localization within cells transfected with the plasmids discussed above. At 72 h post-transfection, cells were fixed using 4% paraformaldehyde followed by staining with polyclonal anti-HBsAg (red), monoclonal anti-KDEL (green) as a marker of ERS, and DAPI (blue) as a nuclear counterstain. HBsAg levels were significantly reduced in those cells that had been transfected with SHB mutant plasmids, in line with the western immunoblotting results (Figures 2 and 3; column 2). S protein was diffusely present throughout the cytosol in HBV-S-WT transfected cells, whereas it was primarily localized to a perinuclear region aligning with the ER in cells transfected with SHB mutant plasmids (Figures 2 and 3). These data indicated that OBI-associated SHB mutant plasmid transfection was associated with lower levels of HBsAg expression, with this mutant protein primarily accumulating within the ER.

### 3.3 OBI-associated SHB mutations induce ERS

**Figure 4:**
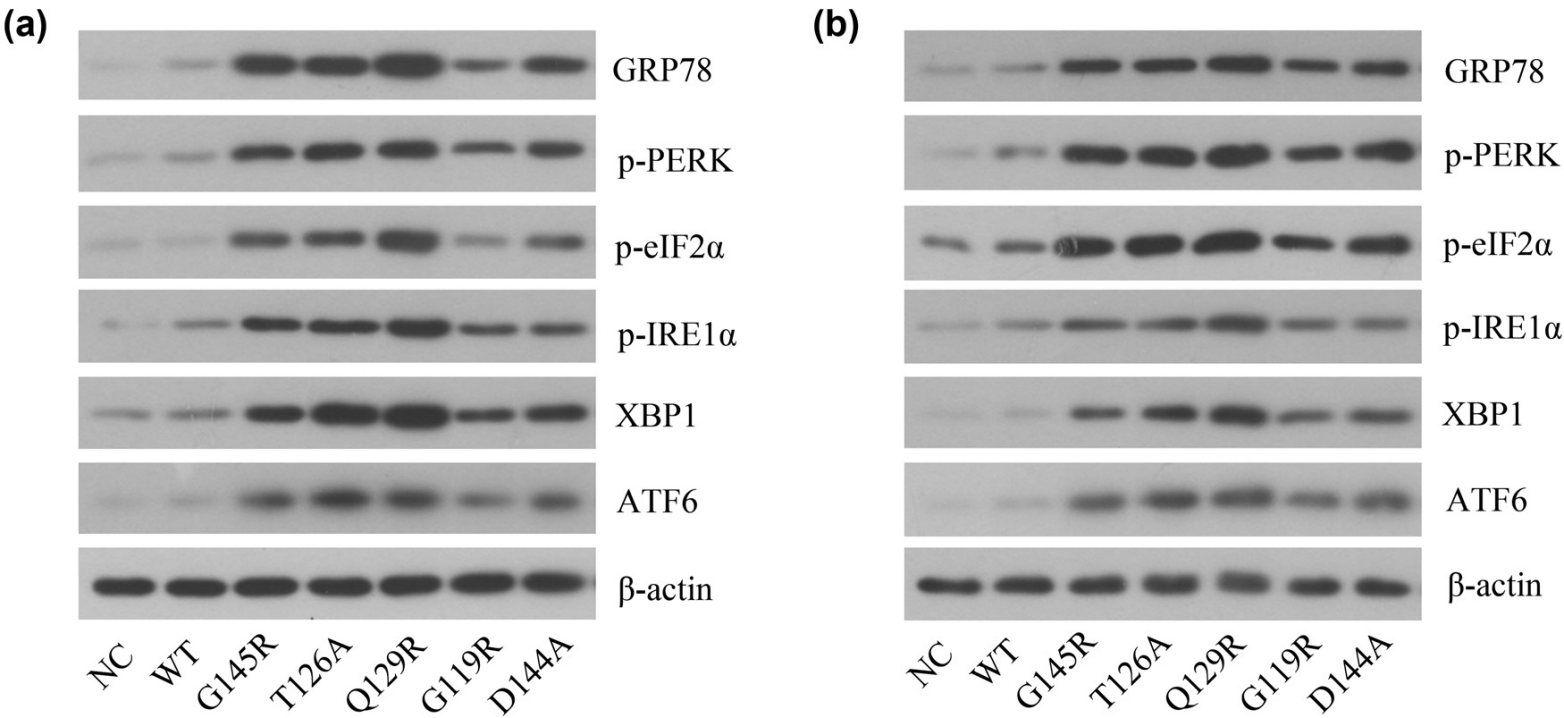
ERS-related protein expression in different SHB plasmid-transfected cells. Western blotting was used to detect ERS-related protein expression levels in HepG2 (a) and Huh7 (b) cells following different SHB plasmid transfections.

The above results demonstrated that transfection with plasmids including SHB mutations resulted in a significant decrease in HBsAg secretion, with this mutated protein primarily accumulating within the ER of transfected cells. In many instances, ERS can be induced by misfolded or unfolded protein accumulation within this organelle [15]. Western blotting was used to assess ERS-UPR related protein levels in these cells, revealing that transfection with these plasmids including OBI-related SHB mutations resulted in an increase in ERS-UPR related protein levels (GRP78, p-PERK/p-eIF2α, p-IRE1α/XBP1, and ATF6) (Figure 4a and b). This suggested that these OBI-associated SHB mutations can promote the induction of ERS.

### 3.4 OBI-related SHB mutations drive EDEM protein expression

**Figure 5:**
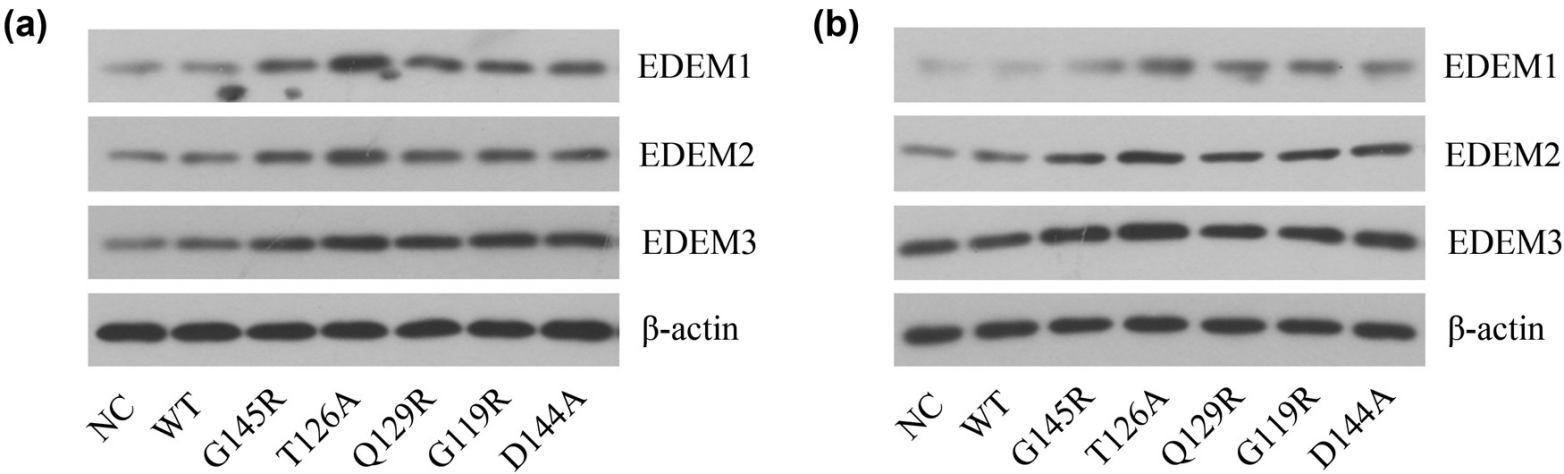
ERAD-associated EDEM protein levels in different SHB plasmid-transfected cells. Western blotting was used to detect ERAD-associated EDEM protein expression levels in HepG2 (a) and Huh7 (b) cells following different SHB plasmid transfections.

ER degradation-enhancing α-mannosidase like proteins (EDEMs) also function as key mediators of the ERAD process, providing a mechanism to differentiate between appropriately folded proteins and those that are misfolded in the context of peptide maturation [20]. In this analysis, relative to cells transfected with WT SHB plasmids, those transfected with plasmids including OBI-related SHB mutations exhibited increased EDEM protein expression (Figure 5). These results suggested that the degradation of ER-related proteins in those cells that have been transfected with these OBI-related SHB mutation plasmids was significantly enhanced.

## 4 Discussion

OBI plays a role in the development of chronic HBV. In one analysis of 32 infants diagnosed with OBI at 7 months of age 25.0% (8/32), 21.9% (7/32), and 7.7% (2/26) were, respectively, diagnosed with HBV infections at 12, 24, and 36 months of age [21]. Immunocompromised patients, individuals co-infected with HIV, hematopoietic stem cell transplant recipients, and patients being treated with immunosuppressive drugs have all been reported to experience HBV reactivation in some instances, with such reactivation being potentially severe and even life-threatening in nearly 20% of cases [22]. The mechanistic basis for OBI, however, remains incompletely understood. The aa99–169 region of HBsAg is present in the MHR, and MHR has a relatively conserved “α” determinant cluster (aa 124–147). Amino acid substitutions in the MHR region can cause conformational changes in the epitope, thereby affecting the hydrophilicity of S proteins and leading to changes in protein properties [11]. Mutations in the HBsAg protein are known to enable this protein to be undetectable while altering its overall antigenicity and immunogenicity and damaging the secretion of virions and HBV replication [10].

Following preS/S gene mutation-containing plasmids transfection, cells primarily exhibit the accumulation of the S and L proteins within the perinuclear ER in a granular distribution pattern, and these cells also present with lower HBV transcript levels and the altered secretion of HBsAg [23]. HBV infection can also drive ERAD pathway activation, in turn reducing HBV envelope protein production. This may help sustain the secretion of low levels of viral particles from these infected cells, ultimately supporting the chronic infection of affected patients [24]. These data suggest that HBsAg production and secretion are closely related to the function of the ER, raising the possibility that OBI pathogenesis may also be closely linked to the induction of ERS.

Biswas et al. [25] previously reported that the transfection of cells with plasmids encoding an OBI-associated mutation in the S gene resulted in a high HBsAg IC/EC ratio, with the accumulation of these surface antigens primarily being evident within cells. Huang et al. [10] further determined that the OBI-associated G119R, Q129R, D144A, and G145R mutations in the S gene were linked to higher IC HBsAg levels, with Lee et al. [26] demonstrating that such mutated HBsAg primarily accumulates within the ER. OBI-associated mutations can also reportedly elevate levels of Ca^2+^ within cells, thereby exacerbating ERS induction and contributing to the increase in reactive oxygen species production that can culminate in the death of affected cells, suggesting a role for ERS in HBV S gene mutation-associated OBI and liver disease progression [26].

For the present study, Huh7 and HepG2 cells were transfected with plasmids encoding WT or mutant SHBs. At 72 h post-transfection, IC and EC HBsAg levels were significantly lower for those cells that had been transfected with plasmids including OBI-associated SHB mutations as compared to cells transfected with plasmids including WT SHBs, suggesting a significant decrease in overall HBsAg expression in the OBI-related mutant group of cells. Moreover, immunofluorescence results suggest that the mutated isoform of HBsAg also primarily accumulated in ER in these transfected cells, in line with prior research [23,26]. It was suggested that the defective S protein was located within the ER and could not be secreted into the cytoplasm. The ER is an important organelle that plays an important role in protein synthesis and is particularly important in the proper folding and modification of proteins secretion. ERS occurs when the accumulation of unfolded or misfolded proteins in the ER which exceeds the ability of protein folding and trigger an UPR. The specific mutation of SHB may lead to the deficiency of S protein secretion, resulting in the accumulation and aggregation of S protein in the ER of hepatocytes. Thus, the homeostasis of the ER was disrupted, which led to ERS and hepatocyte injury. In our research, transfection with OBI-associated SHB mutation-containing plasmids also results in significant increases in the expression of the ER molecular chaperone GRP78. Under normal circumstances, GRP78 mainly binds to the transmembrane proteins of the ER. However, when excessive accumulation of misfolded and/or unfolded proteins occurs in ER, the ER pressure increases and GRP78 is released. It binds to the accumulated protein, preventing the protein from being secreted out of the ER. Thus, the ER UPR is initiated [27]. In addition, we found that transfection with OBI-associated SHB mutation-containing plasmids also results in significant increases in the expression of the ERS-UPR pathway proteins (p-PERK/eIF2α p-IRE1α/XBP1 ATF6). It was suggested that a large number of defective proteins accumulate in the ER with SHBs mutation in the S gene. And misfolded protein accumulation within ER can activate ERS. ERS signals are subsequently transmitted to the cell membrane to induce and exacerbate the UPR [28], which ultimately impairs S protein secretion. UPR contains at least three different mechanisms, including: down-regulate transcription of corresponding genes, reduce the level of protein translation, and reduce the synthesis of new proteins [29]. It also results in the expression of a range of proteins that can help protect cells, including glutathione, GRP94, and GRP78 [30], while also inducing apoptotic death when the underlying stress is not effectively resolved [31]. The UPR can also recognize and ensnare unfolded proteins with EDEMs such that they can be trafficked to the ubiquitin-proteasome degradation via the ERAD pathway [10]. EDEMs are reportedly important mediators of ERAD activity owing to their ability to differentiate between mature and misfolded proteins, performing a quality control function in the context of peptide maturation [20]. We further investigated the expression of EDEMs, and revealed that EDEM protein levels were significantly elevated in those cells that had been transfected with plasmids including SHB mutations. Overall, these data support a model in which surface antigens, when mutated, can accumulate within the ER, thereby driving ERAD-mediated protein degradation through the induction of ERS and UPR responses, then the level of surface antigen secretion is reduced.

## 5 Conclusions

In summary, these results demonstrate that OBI-associated SHB mutations may change the folding process of HBsAg, promote UPR reaction, and reduce the transcription and translation level of HBsAg, thereby decreasing the overall level of HBsAg synthesis. UPR activation can also drive ERAD pathway activation and the transport of the mutated HBsAg protein into the ubiquitination proteasome for degradation such that the secretion level of surface antigen is further decreased, and the level of serum HBsAg are lower, contributing to the occurrence of OBI.
